# Society Meetings

**Published:** 1904-11

**Authors:** 


### SOCIETY MEETINGS.

The Fourth Pan-American Medical Congress, which will convene in Panama the first week in January, 1905, bids fair to be a most delightful mid-winter trip. The delegates will leave this country by the Atlantic, Pacific, and Gulf coasts the last week in December. They will return by the same routes, or will make round trips.

The Public Health Association meeting will take place on the following week in Havana, and those desirous of attending both meetings, can arrange to do so.

There are two routes for the physicians to take from Panama to Havana. The first is by way of Jamaica to Santiago de Cuba by boat and overland by rail to Havana. The second is by water from Panama to Vera Cruz and from there to Havana. The former will probably be the most pleasant trip.

From Havana, the return trip can be made directly north to New York by water or via Miami or Tampa, Florida, or New Orleans. The connections and dates of sailing are now being arranged.

The Panamanian Government has appropriated $25,000 for the scientific sessions and the entertainments. The congress will be held from the second to the sixth of January. The afternoons will be devoted to the scientific sessions and the mornings and evenings to trips and social functions. So far as can be learned, the program in Panama will be a reception on the first day, by President Amador, of the Panama Republic, and the formal opening session of the congress the same evening. On the second day, an excursion to the canal in the morning, meeting of the various sections in the afternoon, and a banquet in the evening; on the third day, an excursion down the bay to Taboga Island, where a Panama breakfast will be served, scientific sessions in the afternoon and a ball in the evening. On the fourth day, an excursion to the United States Army barracks in the morning, section meetings in the afternoon and the formal closing session in the evening. On the fifth day, an excursion to the plantation of the United Fruit Company; and on the afternoon of this day, those of the congresses who intend going to Cuba to attend the meeting of the Public Health Association, will sail for Jamaica, while those who intend going by way of Vera Cruz, or returning home by way of New Orleans or New York, will remain until the following Tuesday.

The secretaries of the sections of the congress for the United States, are: A. H. Doty, New York, Hygiene and Quarantine; Judson Daland, Philadelphia, Medicine; R. Matas, New Orleans, General Surgery; Bert Ellis, Los Angeles, Eye; Hudson Makuen, Philadelphia, Throat; Frederick Jack, Boston, Ear; C. H. Hughes, Saint Louis, Nervous Diseases; George Goodfellow, San Francisco, Military Surgery; John Ridlon, Chicago, Orthopedic Surgery; D. W. Montgomery, San Francisco, Dermatology; C. G. Kerley, New York, Pediatrics; Noble P. Barnes, Washington, Therapeutics; Walter Chase, Boston, Pathology.

Communications from physicians in the United States, interested in these branches, can be sent directly to these different secretaries. Delegates intending to attend the congress, desirous of obtaining information concerning it, should communicate with the secretary of the International Executive Committee in the United States, Dr. Ramon Guiteras, 75 West 55th street, New York City.

---

THE quarterly meeting of the Erie County Medical Association was held at the University Club, Buffalo, on Monday evening, October 10, 1904, under the presidency of C. C. Frederick. The program was as follows: Some studies in metabolism, Herbert H. Glosser. The necessity of systematic examination of the eyes of defectives, F. Park Lewis.

David E. Wheeler was the secretary.

---

THE Mississippi Valley Medical Association held its thirtieth annual meeting at Cincinnati, October 12-13, 1904, at which the following-named officers were elected: president, Bransford

Lewis, Saint Louis; first vice-president, Frank Parsons Norbury, Jacksonville, Ill.; second vice-president, J. H. Carstens, Detroit; secretary, Henry Enos Tuley, Louisville; assistant secretary, John F. Barnhill, Indianapolis; treasurer, S. C. Stanton, Chicago. The next place of meeting is Indianapolis, October, 1905.

---

The Buffalo Academy of Medicine held meetings during the month of October as follows:

A stated meeting of the academy was held Tuesday evening, October 4. The program of the evening was furnished by the surgical section, as follows: (a) The differential diagnosis in some gallbladder conditions, John Parmenter; (b) The question of drainage in operative work on the gallbladder and hepatic ducts, Eugene A. Smith.

*Section on Medicine.*—Tuesday evening, October 11. Program: (a) Essential medication in cardiac diseases, Eli H. Long; discussion by Charles G. Stockton and DeLancey Rochester; (b) Gastric ulcer, A. L. Benedict; discussion by Henry R. Hopkins and F. C. Busch.

*Section on Pathology.*—Tuesday evening, October 18. Program: (a) Two cases of double ureter, bilateral, James A. Gibson; (b) Concerning the accuracy of the hypobromite method for the estimation of urea, Willet H. Mosher, Ph.D.; (c) Practical demonstration of some points in the physiology of the heart, F. C. Busch; (d) Some experiments on the influence of the *x*-ray on the heart-beat and circulation in the frog. William Ward Plummer.

*Section on Obstetrics and Gynecology.*—Tuesday evening, October 25. Program: Fibroid of the ovary, F. W. McGuire; Face presentation, R. L. Banta.

---

The New York State Medical Association held its annual meeting at the New York Academy of Medicine, October 17-20, 1904, at which the following-named officers were elected: president, J. Riddle Goffe, New York; vice-president, Allen A. Jones, Buffalo; treasurer, F. A. Baldwin, New York; secretary, Charles I. Redfield, Middletown.

---

The Medical Association of Central New York held its thirty-seventh annual meeting Tuesday, October 18, 1904. The following program was observed: (1) President's address, Charles A. Vanderbeek, Rochester; (2) Toxins and acute infectious diseases as factors in the etiology of insanity, Frank H. Stephenson, Syracuse; (3) Melancholia and the new plan of treatment, Floyd S. Crego, Buffalo; (4) Tic Douloureux from accessory sinus disease, John O. Roe, Rochester; (5) Pharmacal fraud, Albert L. Hall, Fulton; (6) The magnet in eye work—its use in two cases, (by invitation), Albert C. Snell, Rochester; (7) Quarantine, Frederick H. Sawers, Rochester; (8) Inflammation—its control, J. Henry Dowd, Buffalo; (9) Anomalously distributed pains in vascular and cardiac diseases, Henry L. Elsner, Syracuse; (10) Subject to be announced, John F. W. Whitbeck, Rochester; (11) Pain in the abdomen, Charles E. Congdon, Buffalo; (12) Remarks on muscular insufficiency of the stomach, Nathan W. Soble, Rochester.

---

THE American Public Health Association will hold its thirty-second annual meeting at Havana, Cuba, Monday, Tuesday, Wednesday, Thursday and Friday, January 9, 10, 11, 12 and 13, 1905, under the following administration; president, Carlos J. Finlay, Havana, Cuba; first vice-president, Jesus E. Monjaras, Mexico; second vice-president, William C. Woodward, Washington, D. C.; secretary, Charles O. Probst, Columbus, Ohio; treasurer, Frank W. Wright, New Haven, Conn.

Transportation rates are not yet available, but the fare from New York and return will not exceed $70, and will probably be less. Full information in regard to transportation and hotel rates will be given at an early date.

---

THE Esculapian Club was entertained by Dr. W. G. Bissell at the Markeen at its regular monthly meeting in October. Dr. George B. Stocker read a paper on Ether anesthesia, which was discussed by Drs. Colton, Seltsman, McGuire, Hoffman, Carr, and Congdon.

